# Draft Genome Sequences of Cefepime-Resistant and -Susceptible Escherichia coli Strains and Imipenem-Resistant and -Susceptible Pseudomonas aeruginosa Strains

**DOI:** 10.1128/MRA.00749-21

**Published:** 2021-12-02

**Authors:** Kendra Batchelder, Liz Ward, Elsa Collins, Caitlin Miles, Stefania Palm, Aisha Mohamed, Larry Feinstein

**Affiliations:** a University of Maine at Presque Isle, Presque Isle, Maine, USA; Loyola University Chicago

## Abstract

Draft genome sequences of Escherichia coli and Pseudomonas aeruginosa strains collected from clinical infections were used to determine the prevalence of newly emerging antibiotic resistance genes in Maine. Comparisons between cefepime-resistant and -susceptible E. coli strains and imipenem-resistant and -susceptible P. aeruginosa strains are being conducted.

## ANNOUNCEMENT

Antibiotic-resistant bacteria continue to be a growing health concern (1). Carbapenems have proven to be effective antibiotics, but emerging resistance is developing in *Enterobacteriaceae* due to novel β-lactamases (2). Some resistance mechanisms require multiple genes and molecular regulators working together, while other mechanisms require a single gene (3). Cefepime-resistant and susceptible Escherichia coli isolates and imipenem-resistant and susceptible Pseudomonas aeruginosa isolates were collected from hospitals in Maine to detect antibiotic resistance genes (ARGs) and to explore their interactions through gene transcription network models. Institutional review board approval and ethics committee review were not required for this study, and all software utilized default parameters unless otherwise noted.

Clinical isolates were collected in 2019 to 2020 from St. Joseph’s Hospital (Bangor, ME, USA), Houlton Regional Hospital (Houlton, ME, USA), and Northern Light A. R. Gould Hospital (Presque Isle, ME, USA). Hospitals cultured the microbes on tryptic soy (TS) agar slants and determined cefepime and imipenem resistance through Vitek antimicrobial susceptibility testing (4). The cultures were transferred to TS broth and incubated for 18 h at 37°C. The broth was used to create TS agar streak plates, and pure cultures grown in TS broth were obtained from isolated colonies. Genomic DNA was extracted with a DNeasy UltraClean kit (Qiagen, Germantown, MD), and isolate cultures were stored in 25% glycerol at −80°C.

The extracted DNA was quantified with a Qubit 4 fluorometer (Thermo Fisher Scientific, Waltham, MA, USA) and Qubit double-stranded DNA (dsDNA) HS assay kit (Thermo Fisher Scientific). Sequencing libraries were prepared using a Nextera XT DNA library preparation kit (Illumina, USA) and Nextera Index kit (Illumina), following the manufacturer’s protocol. The standard protocol was used for a total DNA input of 1 ng. Genomic DNA was fragmented using a proportional amount of Illumina Nextera XT fragmentation enzyme. Combinatory dual indexes were added to each sample, followed by 12 cycles of PCR to construct libraries. DNA libraries were purified using AMPure magnetic beads (Beckman Coulter, USA) and were eluted in Qiagen EB buffer. DNA libraries were pooled for sequencing on the Illumina HiSeq X system.

Raw sequencing reads were trimmed and processed using BBDuk (5) with a read quality trimming parameter of 22 for further downstream analysis. The trimmed fastq files were assembled *de novo* using SPAdes v3.12.0 (6) with the –careful parameter. Strains were identified using ContEst16S (7) and BLAST v2.11.0 (8). Completeness and contamination were assessed using CheckM v1.1.2 (9). PGAP v4.11 (10) and RASTtk v1.073 (11, 12) were used for annotation. Draft genome sequence assembly information, cefepime and imipenem resistance phenotypes, infection sources, and statistics are shown in Table 1.

**TABLE 1:** Draft genome sequence information

Isolate	Species	Strain	RP code^*a*^	Source^*b*^	SRA accession no.	GenBank accession no.	No. of reads	Assembly coverage (×)	Genome size (bp)	CheckM completeness (%)	No. of contigs	*N*_50_ (bp)	GC content (%)
BECR1	E. coli	SCU-182	CR	Urine	SRX11095338	JAHVKT000000000	3,142,456	205	4,938,147	99.96	89	249,452	50.698
BECR2	E. coli	SCU-182	CR	Urine	SRX11095339	JAHVKU000000000	3,153,882	206	4,927,284	99.96	95	199,884	50.691
BECR3	E. coli	142	CR	Urine	SRX11095382	JAHVKV000000000	3,570,087	233	5,028,157	99.96	108	188,468	50.468
BECR4	E. coli	ATCC 98082	CR	Urine	SRX11095393	JAHVKW00000000	3,976,641	259	5,158,658	99.96	124	190,962	50.744
BECR5	E. coli	J-8	CR	Urine	SRX11095404	JAHVKX000000000	4,043,723	264	4,803,250	99.96	128	161,135	50.613
BECR6	E. coli	SCU-175	CR	Urine	SRX11095351	JAHVKY000000000	3,746,975	244	5,052,028	99.96	123	151,646	50.331
BECR7	E. coli	ATCC 98082	CR	Urine	SRX11095362	JAHVKZ000000000	2,911,882	201	5,131,702	99.96	86	236,138	50.817
BECR8	E. coli	2547	CR	Urine	SRX11095373	JAHVLA000000000	3,786,128	277	5,026,347	99.65	110	204,108	50.568
BECR9	E. coli	SCU-175	CR	Urine	SRX11095416	JAHVLB000000000	3,191,469	208	5,042,130	99.96	108	149,419	50.356
BECR10	E. coli	2547	CR	Urine	SRX11095424	JAHVLC000000000	2,799,260	183	5,059,171	99.96	114	205,857	50.613
BECR11	E. coli	2547	CR	Urine	SRX11095340	JAHVLD000000000	2,689,543	299	5,179,409	99.96	160	180,896	50.631
HECR1	E. coli	SCU-176	CR	Urine	SRX11095386	JAHVLQ000000000	3,218,163	210	5,037,696	99.96	91	222,504	50.732
HECR2	E. coli	ATCC 98082	CR	Urine	SRX11095387	JAHVLR000000000	3,726,933	243	5,084,267	99.96	119	186,706	50.706
HECR3	E. coli	ATCC 98082	CR	Urine	SRX11095388	JAHVLS000000000	2,175,589	142	5,104,760	99.96	84	190,962	50.779
HECR4	E. coli	EcPF40	CR	Urine	SRX11095389	JAHVLT000000000	3,171,970	207	5,015,846	99.96	100	190,962	50.768
HECR5	E. coli	ATCC 98082	CR	Urine	SRX11095390	JAHVLU000000000	3,387,736	221	5,179,856	99.96	131	174,069	50.751
HECR6	E. coli	ATCC 98082	CR	Urine	SRX11095391	JAHVLV000000000	4,050,093	264	5,094,021	99.96	120	174,069	50.792
HECR7	E. coli	ATCC 98082	CR	Urine	SRX11095392	JAHVLW000000000	3,411,916	223	5,147,765	99.96	146	200,290	50.664
HECR8	E. coli	19-5	CR	Urine	SRX11095394	JAHVLX000000000	3,116,767	203	4,741,301	99.96	173	89,378	50.746
HECR9	E. coli	ATCC 98082	CR	Urine	SRX11095395	JAHVLY000000000	3,339,255	218	5,138,989	99.96	136	141,216	50.714
HECR10	E. coli	ATCC 98082	CR	Urine	SRX11095396	JAHVLZ000000000	2,952,591	193	5,242,776	99.96	134	181,174	50.626
HECR11	E. coli	ATCC 98082	CR	Urine	SRX11095397	JAHVMA000000000	2,687,432	175	5,207,374	99.96	149	174,070	50.713
HECR12	E. coli	ATCC 98082	CR	Urine	SRX11095398	JAHVMB000000000	3,933,434	257	5,152,476	99.96	133	174,071	50.68
HECR13	E. coli	ATCC 98082	CR	Urine	SRX11095399	JAHVMC000000000	3,383,203	221	5,169,424	99.96	155	182,892	50.673
HECR14	E. coli	ATCC 98082	CR	Urine	SRX11095400	JAHVMD000000000	3,341,370	218	5,089,220	99.96	140	186,706	50.686
HECR15	E. coli	ATCC 98082	CR	Urine	SRX11095401	JAHVME000000000	3,096,515	202	5,141,666	99.96	140	191,738	50.682
HECR16	E. coli	ATCC 98082	CR	Urine	SRX11095402	JAHVMF000000000	3,148,766	205	5,265,519	99.96	120	170,618	50.75
HECR17	E. coli	ATCC 98082	CR	Urine	SRX11095403	JAHVMG000000000	3,413,026	223	4,953,345	99.96	224	58,815	50.558
HECR18	E. coli	ATCC 98082	CR	Urine	SRX11095405	JAHVMH000000000	3,353,124	219	5,170,930	99.95	127	183,615	50.716
HECR19	E. coli	ATCC 98082	CR	Urine	SRX11095342	JAHVMI000000000	2,968,960	194	5,173,431	99.34	140	200,157	50.693
HECR20	E. coli	ATCC 98082	CR	Urine	SRX11095343	JAHVMJ000000000	3,873,553	253	5,093,621	99.86	129	186,706	50.729
PIECR1	E. coli	963	CR	Urine	SRX11095369	JAHVNH000000000	2,912,054	190	5,201,590	99.96	144	163,176	50.737
PIECR2	E. coli	ATCC 98082	CR	Urine	SRX11095369	JAHVNI000000000	3,718,512	243	4,985,684	99.96	103	161,044	50.747
BECS1	E. coli	EcPF5	CS	Urine	SRX11095371	JAHVLE000000000	3,580,022	233	5,116,067	99.96	89	320,010	50.438
BECS2	E. coli	2547	CS	Urine	SRX11095374	JAHVLF000000000	3,578,524	233	5,063,189	99.96	115	198,464	50.686
BECS3	E. coli	ATCC 98082	CS	Urine	SRX11095375	JAHVLG000000000	3,824,172	249	5,098,202	99.96	129	186,506	50.73
BECS4	E. coli	J-8	CS	Sepsis	SRX11095376	JAHVLH000000000	3,152,287	206	5,008,583	99.96	121	145,273	50.657
BECS5	E. coli	CFSAN061768	CS	Urine	SRX11095377	JAHVLI000000000	3,804,289	248	5,055,899	99.96	116	178,938	50.524
BECS6	E. coli	EcPF5	CS	Urine	SRX11095378	JAHVLJ000000000	3,709,488	242	4,840,683	99.96	64	400,644	50.627
BECS7	E. coli	SCU-123	CS	Urine	SRX11095379	JAHVLK000000000	2,911,882	190	5,082,726	99.96	70	225,911	50.54
BECS8	E. coli	ATCC 98082	CS	Urine	SRX11095380	JAHVLL000000000	3,786,128	247	5,022,401	99.65	100	203,898	5.0447
BECS9	E. coli	CP66-6	CS	Urine	SRX11095381	JAHVLM000000000	2,702,646	176	4,725,166	99.96	104	198,618	50.642
BECS10	E. coli	EcPF7	CS	Urine	SRX11095424	JAHVLN000000000	2,790,938	182	5,059,171	99.96	114	205,857	50.652
BECS11	E. coli	SCU-122	CS	Urine	SRX11095384	JAHVLO000000000	2,689,543	175	5,179,409	99.96	160	180,896	50.629
HECS1	E. coli	SCU-306	CS	Urine	SRX11095344	JAHVMK000000000	3,305,028	216	5,163,402	99.96	131	346,063	50.494
HECS3	E. coli	EcPF5	CS	Urine	SRX11095346	JAHVMM00000000	3,193,975	208	5,114,414	99.96	163	186,542	50.365
HECS4	E. coli	SCU-123	CS	Urine	SRX11095347	JAHVMN000000000	2,661,456	174	5,085,659	99.96	73	385,686	50.535
HECS5	E. coli	SCU-176	CS	Urine	SRX11095348	JAHVMO000000000	3,820,871	249	5,133,578	99.96	109	239,178	50.513
HECS7	E. coli	RM13322	CS	Urine	SRX11095350	JAHVMQ000000000	2,998,049	196	4,630,927	99.96	73	197,232	50.813
HECS9	E. coli	BCE049	CS	Urine	SRX11095353	JAHVMS000000000	4,898,315	319	5,363,395	99.96	122	230,516	50.4
HECS10	E. coli	EcPF5	CS	Urine	SRX11095354	JAHVMT000000000	3,106,771	203	5,162,231	99.96	91	270,402	50.386
PIECS1	E. coli	CFSAN067215	CS	Urine	SRX11095372	JAHVNK000000000	3,347,521	218	5,155,668	99.96	125	186,738	50.495
PIECS2	E. coli	SCU-176	CS	Urine	SRX11095406	JAHVNL000000000	2,656,411	173	5,071,746	99.96	110	155,839	50.671
PIECS3	E. coli	J-8	CS	Urine	SRX11095407	JAHVNM000000000	3,463,705	226	5,176,436	99.96	113	205,377	50.491
PIECS4	E. coli	J-8	CS	Urine	SRX11095408	JAHVNN000000000	3,381,025	221	5,178,301	99.96	128	202,554	50.455
PIECS5	E. coli	HB37	CS	Urine	SRX11095409	JAHVNO000000000	2,869,902	187	5,252,708	99.96	139	174,862	50.318
PIECS6	E. coli	EcPF5	CS	Urine	SRX11095410	JAHVNP000000000	3,658,961	239	4,977,116	99.96	112	266,915	50.479
PIECS7	E. coli	EcPF5	CS	Urine	SRX11095411	JAHVNQ000000000	3,338,872	218	5,122,956	99.96	113	202,590	50.52
PIECS8	E. coli	ATCC 98082	CS	Urine	SRX11095412	JAHVNR000000000	3,529,069	230	4,941,286	99.96	120	182,999	50.514
PIECS9	E. coli	HB37	CS	Urine	SRX11095413	JAHVNS000000000	3,351,475	219	5,066,796	99.96	97	267,073	50.415
PIECS10	E. coli	K-15KW01	CS	Urine	SRX11095414	JAHVNT000000000	2,604,799	170	5,109,496	99.96	99	239,205	50.428
HPAR1	P. aeruginosa	PA0750	IR	Urine	SRX11095355	JAHVMU000000000	3,005,377	143	7,064,446	99.68	172	253,444	65.734
HPAS1	P. aeruginosa	PA0750	IS	Urine	SRX11095356	JAHVMV000000000	3,327,641	158	6,350,645	99.68	83	340,986	66.417
HPAS3	P. aeruginosa	PA0750	IS	Urine	SRX11095357	JAHVMW00000000	3,078,500	201	5,022,850	99.96	166	199,572	50.633
HPAS4	P. aeruginosa	PA0750	IS	Urine	SRX11095358	JAHVMX000000000	2,873,871	137	6,877,445	99.68	193	246,992	65.782
HPAS5	P. aeruginosa	PA0750	IS	Sputum	SRX11095359	JAHVMY000000000	2,169,798	103	6,628,691	99.68	149	270,441	65.959
HPAS6	P. aeruginosa	PA0750	IS	Urine	SRX11095360	JAHVMZ000000000	3,066,044	146	6,517,951	99.68	139	382,386	66.143
HPAS7	P. aeruginosa	PA0750	IS	Urine	SRX11095361	JAHVNA000000000	2,415,639	115	6,325,701	99.68	111	275,865	66.37
HPAS8	P. aeruginosa	PA0750	IS	Sputum	SRX11095363	JAHVNB000000000	3,702,607	176	6,645,144	99.68	131	299,638	65.962
HPAS9	P. aeruginosa	PA0750	IS	Sputum	SRX11095364	JAHVNC000000000	3,004,365	143	6,835,785	99.57	118	268,894	66.071
HPAS10	P. aeruginosa	PA0750	IS	Urine	SRX11095365	JAHVND000000000	2,448,815	117	6,665,979	99.68	140	145,624	66.125
HPAS11	P. aeruginosa	PA0750	IS	Sputum	SRX11095366	JAHVNE000000000	3,325,036	158	6,506,715	99.68	120	209,648	65.928
HPAS12	P. aeruginosa	PA0750	IS	Urine	SRX11095367	JAHVNF000000000	2,407,931	115	6,514,459	99.57	121	219,789	66.249
HPAS13	P. aeruginosa	PA0750	IS	Urine	SRX11095368	JAHVNG000000000	3,066,928	146	6,716,233	99.68	112	246,977	66.103
PIPAS2	P. aeruginosa	PA0750	IS	Urine	SRX11095415	JAHVNU000000000	3,464,349	165	7,026,770	99.68	146	245,125	65.908
PIPAS3	P. aeruginosa	PA0750	IS	Urine	SRX11095417	JAHVNV000000000	2,722,515	103	6,475,538	99.68	128	241,111	66.175
PIPAS4	P. aeruginosa	A681	IS	Urine	SRX11095418	JAHVNW00000000	3,142,506	150	7,001,199	99.68	150	286,909	65.88
PIPAS5	P. aeruginosa	PA0750	IS	Urine	SRX11095419	JAHVNX000000000	2,901,172	138	6,855,447	99.68	112	232,113	66.025
PIPAS6	P. aeruginosa	PA0750	IS	Urine	SRX11095420	JAHVNY000000000	2,559,019	122	6,354,776	99.68	85	308,015	66.391
PIPAS7	P. aeruginosa	PA0750	IS	Urine	SRX11095421	JAHVNZ000000000	2,722,184	130	6,834,721	99.57	115	280,449	66.073
PIPAS8	P. aeruginosa	SCU-175	IS	Urine	SRX11095422	JAHVOA000000000	2,622,042	171	4,988,358	99.96	119	186,591	50.591
PIPAS9	P. aeruginosa	PA0750	IS	Urine	SRX11095423	JAHVOB000000000	2,984,944	130	6,466,828	99.68	92	250,133	66.293
BPAS1	P. aeruginosa	PA0750	IS	Sepsis	SRX11095385	JAHVLP000000000	2,434,059	159	6,366,539	99.63	103	260,454	66.328

a RP, resistance phenotype; CR, cefepime resistant; CS, cefepime susceptible; IR, imipenem resistant; IS, imipenem susceptible.

b Source, infected region from which the isolate was collected.

The contigs were uploaded to the Resistance Gene Identifier (RGI) at the Comprehensive Antibiotic Resistance Database (CARD) (13) to identify ARGs and were analyzed using ISEScan (14) to detect insertion elements. Plasmid contigs were assembled from raw reads using plasmidSPAdes (15), and ARGs present on the plasmids were determined with the RGI (13). A total of 5,079 ARGs, 410 plasmids, and 1,628 insertion sequences were found in the 87 isolates.

### Data availability.

The raw reads and assembled contigs are available under NCBI BioProject number PRJNA732585, with the accession numbers listed in Table 1.
